# The relationships of psychological capital and influence regulation with job satisfaction and job performance

**DOI:** 10.1371/journal.pone.0272412

**Published:** 2022-08-09

**Authors:** Mateusz Paliga, Barbara Kożusznik, Anita Pollak, Elżbieta Sanecka

**Affiliations:** Institute of Psychology, University of Silesia in Katowice, Katowice, Poland; Taiyuan University of Science and Technology, CHINA

## Abstract

**Background and aims:**

The individual difference predictors of positive work attitudes and behaviors have been widely investigated in the field of positive organizational scholarship. However, to date, integrating studies linking positive psychological resources, such as Psychological Capital and influence regulation, with positive organizational outcomes are still scarce. Thus, the main aim of the present study was to examine the relationships of Psychological Capital and influence regulation with job satisfaction and job performance both at the individual and team levels.

**Methods:**

Within the cross-sectional multi-source research involving both team leaders and team members from 34 different teams, we examined the relationships of Psychological Capital and influence regulation with job satisfaction and job performance. The relationships of the study variables were based on the positive organizational behavior and the broaden-and-build theory of positive emotions, which suggest the positive relationships of distinct positive psychological resources with positive work outcomes. Accordingly, in addition to the widely accepted concept of Psychological Capital (PsyCap), we employed and analyzed the complimentary construct of influence regulation (i.e., the ability to intentionally share social influence with others in the workplace) both at the individual and group levels.

**Results:**

The results of hierarchical linear modeling with 304 individuals from 34 teams from a diverse sample of Polish employees indicated that team members’ PsyCap was positively linked to individual-level job satisfaction and two facets of job performance, i.e. creative performance and in-role performance. In contrast, no relationship was found between influence regulation and job satisfaction or job performance at both levels of analysis.

**Conclusion:**

With regard to positive interpersonal resources, the findings highlight the role of PsyCap in predicting job satisfaction and job performance and broaden the understanding of positivity in the workplace by introducing the construct of influence regulation. Also, based on the study results, managerial implications are discussed.

## Introduction

Human resources are critical to achieving a sustainable competitive advantage, especially as the pace of change affecting organizations increases [1,2]. Within the paradigm of positive organizational behavior, the source of employee and organizational development consists of groups of resources that operate on different levels in an organization and have to be strengthened. Accordingly, positive individual traits and states constitute psychological resources at the individual level. They lead to valuable subjective experiences, such as well-being and satisfaction. At the group level, these resources are related to positive organizational behavior, which is associated with responsibility, altruism, kindness, tolerance, and work ethic [3].

Although positivity in the teamwork setting is not fully understood [4], to our knowledge the majority of the research has been carried out only at the individual level, focusing on employees and neglecting other agents in a teamwork environment, namely, the leader and the team as a whole. However, it is the teamwork and favorable interactions among team members that generate positive affect [5] and bring benefits [6], such as promoting more productive work behavior [7,8] and increasing well-being at work [9,10]. With this in mind, in the current study we adopt Fredrickson’s [11] broaden-and-build theory and Luthans’s [8] positive organizational behavior to carry out a multilevel analysis of the relationships of Psychological Capital and influence regulation with job satisfaction and job performance (i.e., in-role performance and creative performance). This choice of particular variables is due to the fact that leadership influence and employee’s Psychological Capital are widely accepted as salient determinants of employees’ attitudes and behaviours [9]. As this is a preliminary study, we investigate only Psychological Capital and influence regulation, which were considered as essential for employees’ job satisfaction and performance from the perspective of positive psychology, without taking into account potential moderating or mediating variables, reflecting the characteristics of organization, team, leader or team member. Moreover, we consider influence regulation to be an instrument that enhances and reinforces the positivity of PsyCap. Thus, by examining the relationships of Psychological Capital and influence regulation with job satisfaction and job performance at both the individual and team levels, the present study contributes to the research efforts aimed to better understand which positive psychological resources and to what extent might contribute to positive workplace outcomes. As the expected relationships were based on positive organizational behavior, broaden-and-build theory of positive emotions, and Conservation of Resources theory, our results might help to better understand how these associations could shape both at the individual and team levels and bring some managerial implications for enhancing positive work outcomes in teams and among individual employees. The novelty of our study is the inclusion of influence regulation in a resource bundle with PsyCap and the adoption of multi-source report collection and multilevel analysis. To our knowledge, this is one of the first studies to take this approach in the investigation of relationships between individual- and group-level positive resources and job satisfaction and job performance (i.e., in-role performance and creative performance).

## Theoretical background

### Psychological capital

Within the field of positive psychology [12] and subsequent positive organizational behavior [8], Psychological Capital (PsyCap) has been conceptually identified by Luthans and colleagues as “an individual’s positive psychological state of development and is characterized by: (1) having confidence (self-efficacy) to take on and put in the necessary effort to succeed at challenging tasks; (2) making a positive attribution (optimism) about succeeding now and in the future; (3) persevering toward goals and, when necessary, redirecting paths to goals (hope) in order to succeed; and (4) when beset by problems and adversity, sustaining and bouncing back and even beyond (resilience) to attain success” [13, p. 3]. Based on psychological resource theory, the PsyCap dimensions share common mechanistic processes, which makes PsyCap a multidimensional [14] second-order core construct [15]. Ample empirical evidence supports its higher-order nature [e.g., 16–18].

Luthans, Youssef and Avolio [19] indicate that PsyCap is a resource that goes beyond both human capital (experience, knowledge, abilities, and possibilities) and social capital (relationships and networks). It is a malleable resource that can be developed to change employee behavior and shape desirable attitudes [19]. Empirical evidence is mounting that PsyCap is strongly related to employee attitudes, including job satisfaction, organizational commitment, and psychological well-being at work [9]. In addition, research results suggest that PsyCap is related to employee behaviors. More specifically, PsyCap has a positive relationship with organizational citizenship behavior [20,21] and overall job performance [8], both objective and self-reported [15], as well as task and contextual performance [22].

### Influence regulation

Considering social influence as capital [23], it is essential to build and maintain relationships that are either valuable resources or allow creating new work teams that can fulfil shared interests [24,25]. In this context, influence regulation [26], rooted in organizational and social psychology, might be proposed as a complementary construct to PsyCap, reflecting positive relational resources in the workplace. We conceptualize influence regulation as the ability to regulate one’s influence in the workplace in order to adequately fulfil the requirements of a situation, which—similarly to PsyCap—is changeable, i.e., can be developed and trained [26]. Because influence regulation particularly refers to influence withdrawal [27], those who regulate their influence can remain silent even though they could take part in a conversation, wait through a break in a conversation, abstain from commenting, and can diminish their importance in a conversation [26,27]. Also, people who regulate their influence arrange space for others to show their contribution. Hence, they protect those who speak from verbal attacks, keep eye contact with an interlocutor, and approve of other people’s ideas [27]. If there is an individual or team that can effectively manage the situation and obtain better results [27], influence regulation allows the influence to be shared, thus making it possible to profoundly use the knowledge and skills of this team member or the team as a whole [28]. Hence, influence regulation involves the ability to accept one’s vulnerability, despite the shame of not being a dependable individual. This ability, in turn, leaves space for growth and positive development of the self and others [29]. In the organizational setting, the process of regulating influence manifests in social interactions through reducing one’s meaning in a team and offering space for other team members. At work, reducing one’s meaning relies on deliberately reducing the tendency to interrupt, comment, and block the activity of others, e.g. during a conversation in a team. These behaviors result from the belief that the actions of others will yield better results. Offering space to others has more active character and relies on improving the communication style and the conditions in which conversations occur (e.g., protecting against verbal attacks, active listening, involving all team members in discussions) [26].

Existing research confirms that influence regulation is related to a wide range of positive outcomes in the workplace both for team members and team leaders, including high social competences and positive relationships with others [30], emotional acceptance of a managing role [31], high development potential and effectiveness in management work [32], and organizational effectiveness in the form of effective functioning in knowledge organizations [29,32]. In addition, leaders who can regulate their influence play a crucial role in the team setting because they are role models for other individuals and their teams [33,34]. The importance of leaders stems from their function, which in modern organizations consists of encouraging subordinates to achieve greater independence and personal development [26,35]. Consequently, they create a work environment where every person can master beneficial skills and gain positive experience [36,37]. Because managers who use influence regulation show a more positive side of themselves than managers who withhold this influence (e.g., they reduce their meaning by abstaining from commenting and arrange space to make it comfortable for conversation participants), they can lead to open communication and trust building [29], as well as increased team member wellbeing and greater team effectiveness [38,39].

### Job performance and job satisfaction

With regard to job performance, we followed Campbell, McHenry, and Wise’s [40] suggestion of defining it in terms of observable and measurable behavior over which the employee has control, in contrast to performance outcomes [6]. Hence, we operationalize job performance by using two categories: in-role performance—reflecting tasks that are formally included in the job description [41]; and creative performance—which refers to the creation of a valuable, useful new product, service, idea, procedure, or process by individuals who work together in a complex social system [42]. Job satisfaction was defined as a positive or negative work attitude, which comprises knowledge, evaluative judgments, and opinions about work (the cognitive component), emotions and feelings about work (the affective component), and individual predispositions and manifestations of employee’s actions related to performed work (the behavioral component) [43].

### The broaden-and-build theory

According to Fredrickson’s [11] broaden-and-build theory, positive emotions are not mere states experienced by individuals, but rather means to achieve optimal functioning, psychological growth, and improved well-being. There are two principles of the theory. First, the broaden hypothesis holds that positive emotions are able to momentarily expand people’s attention and thought-action repertoires, thus enabling them to draw from a wider range of precepts or ideas. Second, the build hypothesis states that, due to experiencing positive emotions, people are encouraged to grow and build enduring physical, intellectual, psychological, and social resources [44]. Considering the theoretical background of broaden-and-build theory, we argue that PsyCap and influence regulation are durable positive resources stemming from positivity. As such, they are long-term manifestations of optimal functioning that go beyond momentary states of pleasure [45]. Luthans and colleagues [46] claim that a positive resource must be based on both theory and research, have a valid measure, be open to development, and have an impact on employee performance. Regarding influence regulation, the construct is grounded in the situational theories of management [47], Lewin’s [48], spontaneous “power game” in organizations, and Kożusznik’s [49] team subjectivity in organizations. There are two valid instruments to measure it [27,50], and both list a set of behaviors that can be developed, trained, and coached [26]. We think that individuals who can regulate their influence serve as examples of socially integrated people who flourish from positivity [45] and are essential in achieving success in a teamwork setting. Regarding PsyCap as a positive resource, the construct stems directly from positivity [51] and positive organizational behavior [8]. Moreover, the fact that unambiguously positive resources, such as efficacy, hope, optimism, and resiliency, constitute the construct leads to the conclusion that PsyCap is a fundamentally positive psychological resource [52].

A crucial question is whether the resources can enhance job satisfaction and job performance. According to Hobfoll’s [53] Conservation of Resources theory, individuals endeavor to obtain, retain, protect, and foster resources that have symbolic or instrumental value to them. Resources aggregate in caravans [54], meaning that one resource usually forms a bundle with another. We argue that PsyCap and influence regulation are examples of combined resources. The positive psychological resources that make up PsyCap, especially self-efficacy and resiliency, are likely to help individuals experience vulnerability without shame [29], whereas hope and optimism make it possible to see the good and efficiency in others, thus enhancing the regulation of influence. Hobfoll [54] argues that the more resources one has, the easier it becomes to gain new resources in the future, and this gain increases motivation and wellbeing. Furthermore, Fredrickson and colleagues [55] claim that resourceful people can effectively meet life’s challenges, seize its opportunities, and become successful, healthy, and happy. Ample empirical evidence confirms this notion. According to [56], positive feelings are related to individual and organizational performance through complex pathways (e.g., via enhanced creativity and positive relationships). Furthermore, Losada and Heaphy [57] provided support for the relationship between positivity among team members and high performance. The authors showed that teams working in a positive work climate were able to achieve greater results. Additionally, research results [58] suggest that team members’ solution-focused utterances enhance team positivity, and that this relationship is reinforced by taking turns speaking. Because these speaker switches are theoretically related to involving all team members in discussions, which is the essence of influence regulation, provide an important argument for the positive character of influence regulation [58]. Moreover, as Lee’s team [59] noted in their study on empowering leadership and task performance, the relationship between them is curvilinear, which means that too much and too little empowerment can hinder performance. Hence, we argue that both team members’ and team leaders’ influence can impact job performance because it is based on a conscious decision about whose potential best fits the requirements of a situation, and when not to allow others to take over the responsibility because they lack the necessary resources and could be overwhelmed by the requirements. Lastly, according to Froman [60], the essence of positive psychology is the notion that there is high performance and satisfaction in everyone’s future. Based on this, we postulate that the positive resources of PsyCap and influence regulation will have positive attitudinal and behavioral consequences.

## The current study

**Fig 1:**
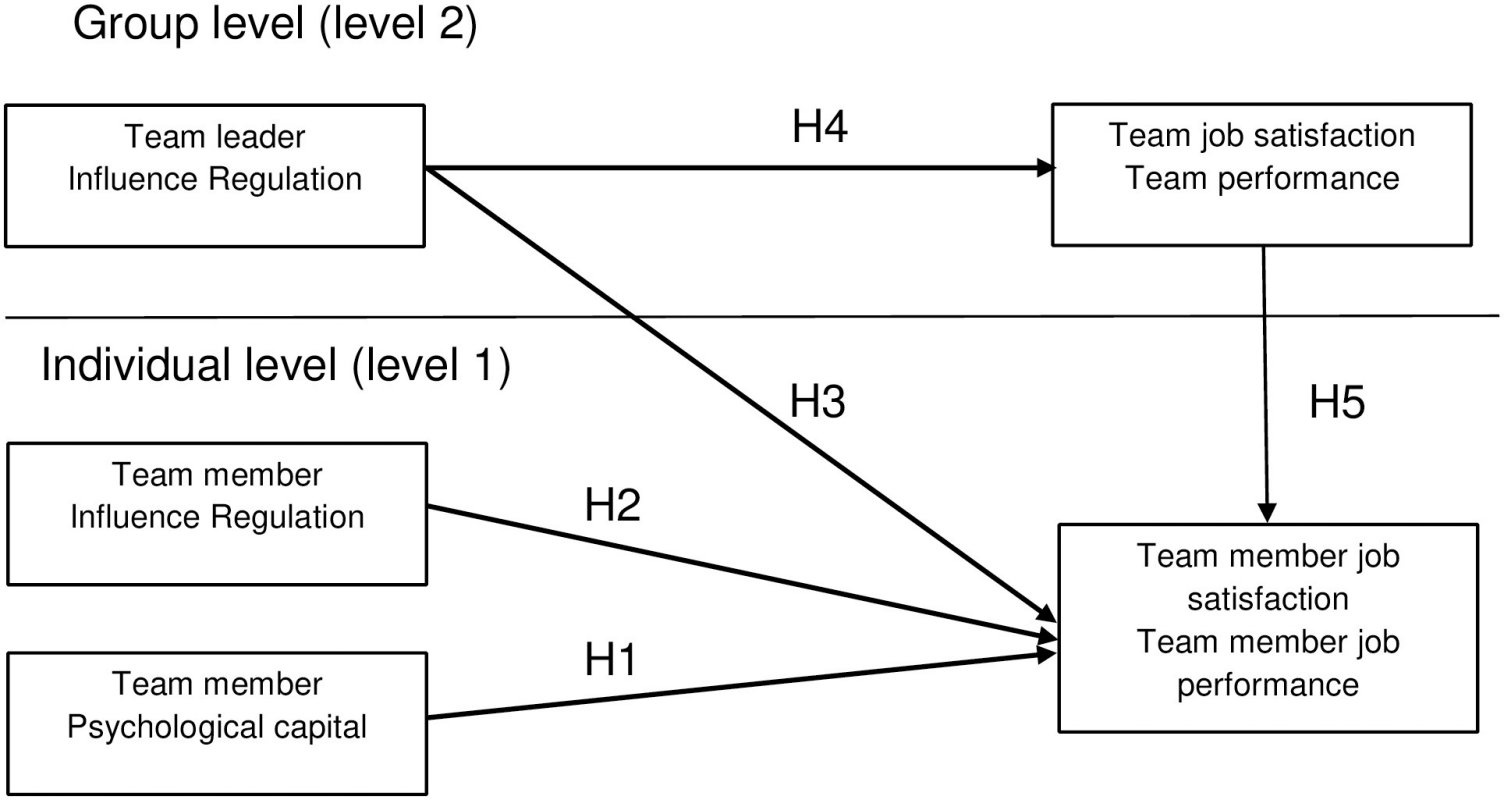
Proposed multilevel research model.

The aim of this study is to investigate the relationships of positive psychological resources in the form of Psychological Capital and influence regulation with positive workplace outcomes, such as job satisfaction and job performance. The relationships between the study variables are analyzed on both the individual (1) and group (2) levels. The individual-level variables were reported by team members and include team members’ characteristics (i.e., PsyCap and influence regulation), along with their general job satisfaction and job performance, including in-role performance and creative performance. In turn, the group-level variables were assessed by the team leader and comprise the team leader’s influence regulation, along with job satisfaction and job performance in the work team. Fig 1 presents a graphical depiction of the multilevel associations among the variables analyzed in the present study. As our study was not focused on investigating the relationships between demographic variables and the main study variables and, based on the previous studies, we expected that the demographic variables in our study would be unimportant or have very little relationships with the main study variables, we did not include demographic variables as a covariates in our study.

The review of existing literature and previous studies suggests that the direct positive relationships between PsyCap and influence regulation with both job satisfaction and job performance could be expected. Regarding PsyCap, there is strong empirical evidence of its positive relations with beneficial employee attitudes [19], including job satisfaction [9]. Moreover, investigations show that PsyCap is related to both overall and contextual job performance [8,22]. Tenney, Poole, and Diener [56] claim that positivity relates to individual performance through diverse pathways, creativity being one of them. This was supported by [57], who showed that team members experiencing a positive work climate could achieve better results. Based on this research, we formulated the following hypothesis:

Hypothesis 1: Individual-level team member’s PsyCap will be positively related to individual-level job satisfaction and job performance (i.e., in-role performance and creative performance).

Concerning influence regulation, its theoretical premise is that the decision of who should take actions to answer the requirements of a situation is based on the assessment of everyone’s possessed resources. The decision is then followed by conscious meaning reduction and space offering of other team members (including the leader), which allow the individual with the most potential to complete the task successfully. Hence, we predict that team members’ and team leaders’ influence regulation will positively relate to job satisfaction and performance. In fact, research shows that influence regulation is advantageous for individuals in a team and their leaders, and the benefits of influence regulation include the presence of positive relationships with others [30], effectiveness in management work [32], and effective functioning in organizations [29,32]. Finally, because influence regulating managers cooperate positively with their subordinates [26], they can increase the well-being and effectiveness of team members [38,39]. Based on these premises, we formulated the following hypotheses:

Hypothesis 2: Individual-level team member’s influence regulation will be positively related to individual-level job satisfaction and job performance (i.e., in-role performance and creative performance).
Hypothesis 3: Group-level team leader’s influence regulation will be positively related to individual-level job satisfaction and job performance (i.e., in-role performance and creative performance).
Hypothesis 4: Group-level team leader’s influence regulation will be positively related to group-level team job satisfaction and team performance.

Lastly, due to the multilevel character of the present study, we expect positive relationships between group-level team job satisfaction and performance and individual-level team member job satisfaction and performance. We base our expectation on the assumption that team members’ job satisfaction and performance are perceived and assessed similarly by the members and their leader who supervises the team actions and attitudes. If so, the multi-source assessment of these phenomena should be positively related. Hence, we formulated the following hypothesis:

Hypothesis 5: Group-level team job satisfaction and team performance will be positively related to individual-level team member’s job satisfaction and job performance (i.e., in-role performance and creative performance).

## Materials and methods

### Participants and procedure

The study is part of a European research project on sustainable wellbeing at work (BELASOS: PSI 2015-64862-R (MINECO FEDER) *Job characteristics and human resource practices as antecedents of sustainable wellbeing at work at different career stages*. Coordinating unit—University of Valencia). The study was approved by the Ethics Committee of the University of Valencia on December 10, 2012 (decision number 111354632059685). In Poland, compliance with national laws and regulations was confirmed by the Ethics Committee of the University of Silesia in Katowice (decision number KEUS.19/04.2020). The study was conducted according to the recommendations of the National Science Centre in Poland regarding studies involving human participation.

Data were collected separately from employees and managers managers recruited from organizations of various sizes operating in the services (e.g., postal administration), industry (e.g., a coal mine), and education (e.g., a university) sectors located in the southern part of the country. We advertised the research to the companies during face-to-face meetings and phone calls. The first contact was made with the general manager or the human resources manager. In a first meeting, researchers explained their project, objectives, time required, and procedure. Then, if the contact person agreed, an invitation was sent to all staff to participate in the study. Participation in the study was voluntary, and subjects could refrain from taking part during the course of the research. Individuals did not receive any financial reward. Participants were ensured that their data would be encoded and processed confidentially. Respondents completed questionnaires in groups during working hours. After providing informed consent, all respondents completed self-description questionnaires. Managers completed the questionnaires to measure influence regulation and the estimated outcome variables in the work team (job satisfaction and job performance). Then, employees assessed their influence regulation and filled out questionnaires for the assessment of PsyCap and the outcome variables (job satisfaction and job performance). The scales used to measure job satisfaction and job performance (besides a 3-item measure of job satisfaction applied at the organizational level) were translated into Polish by the authors. The research group included 338 respondents, 304 employees (89.84%) and 34 managers (10.06%). Teams with at least two employees qualified for the final analysis.

In the group of employees, there were 241 (79.3%) women and 52 men (17.1%); eleven people did not indicate their gender (3.6%). Respondents’ age was classified into three groups: 54 respondents (17.8%) were between 18 and 35 years old, 148 (48.7%) were between 35 and 50 years old, 95 (31.3%) were over 50 years old, and seven did not provide an answer (2.3%). Among the respondents, 157 (51.6%) had completed master’s degrees, 133 (43.8%) had completed secondary education, seven (2.3%) had attended vocational school, and one person had finished primary school (.3%). One participant (.3%) had other degrees, and four of them did not provide an answer. Just over half the sample of managers (55.9%) was female; men constituted 41.2%, and one did not indicate gender (2.9%). Twenty respondents (57.1%) were between 35 and 50 years old, nine were over 50 years old, and five were between 18 and 35 years old. Most of them, 28 people (82.4%), had completed a master’s degree, five (14.7%) had completed secondary education, and one did not provide an answer.

### Measures

#### Psychological capital

Participants completed The Psychological Capital Questionnaire (PCQ-12) [46], which assessed their psychological capital on a 6-point rating 12-item scale ranging from *strongly disagree* (1) to *strongly agree* (6). A sample item is “*Right now I see myself as being pretty successful at work*”. The estimated reliability was *α* = .89.

#### Influence regulation

We measured influence regulation with the Influence Regulation and Deinfluentization Scale (*DEI-beh*) [50]. The scale consists of 20 items measured on a 5-point rating scale from *never* (1) to *always* (5), and it captures the individual’s tendency to regulate influence in the organizational context. A sample item is “*I am able to remain silent*, *although I could take part in a conversation*.” The variable was measured from two sources at two levels of analysis; hence, the team members indicated their influence regulation while the team leaders described theirs. The estimated reliability was *α* = .94 at the individual level and *α* = .75 at the group level.

#### Job performance

To measure performance-related variables at the individual level, we used two separate self-reported scales. Participants completed a three-item In-role Performance Scale [41], indicating the degree to which they complete the tasks formally included in the description of their job. The items were measured on a 7-point rating scale ranging from *strongly disagree* (1) to *strongly agree* (7). A sample item was “*I adequately complete assigned duties*”. The reliability of the scale was *α* = .67. Participants also completed the Creative Performance Scale [61]. The three-item scale indicates the degree to which the employee’s work is original and practical, adaptive and practical, and creative. The items were measured on a 7-point rating scale ranging from *strongly disagree* (1) to *strongly agree* (7). A sample item was “*I am practical at work*. *I develop ideas that are useful to the organization*” The reliability of the scale was *α* = .80.

#### Team performance

General job performance at the group level was assessed by the team leader, who estimated general performance, job quality, and the level of achievement in a team or organizational unit in the past year. The leader answered three questions adapted from Jehn and colleagues’ [62] “members’ perceived group performance scale” using a 5-point scale ranging from 1 *definitely bad* (1) to *definitely good* (5). The three individual items (e.g., “*How well do you think your work team performs*?”) were summed to obtain the total score on team performance. The measure demonstrated satisfactory internal consistency (α = .75).

#### Job satisfaction

To assess team members’ overall job satisfaction, we employed the Job Satisfaction Scale [63]. The nine items were measured on a 7-point scale ranging from *very dissatisfied* (1) to *very satisfied* (7). Sample items included satisfaction with one’s “*Freedom to choose your own method of working*” and “*Physical working conditions*”. The reliability of the scale was α = .85 at the individual level.

The general level of job satisfaction in work teams was rated by the team leaders, who completed a 3-item measure assessing the members’ satisfaction with their job in general, the social climate and relations in their work unit, and salary and additional remuneration. Leaders filled out the questionnaire using a 7-point scale ranging from *very dissatisfied* (1) to *very satisfied* (7). The scale had sufficient internal consistency (*α* = .75).

## Results

The statistics were calculated on the database uploaded to Open Science Framework. The database is available under the following address: https://osf.io/7qfcw/?view_only=a4d5f7a8726841d7b0a94f87b7b86b67.

Means, standard deviations, and zero-order correlations among the study variables at both levels of analysis are reported in Table 1. At the individual level, PsyCap was positively related to team members’ job satisfaction and different aspects of job performance, and individual-level team member’s influence regulation was positively related to individual-level job satisfaction and creative performance. In addition, team members’ influence regulation showed a positive correlation with team members’ psychological capital, suggesting that both constructs might, in part, empirically overlap, sharing the same core of positivity. At the team level, team leaders’ influence regulation was unrelated to group-level job satisfaction and job performance. Finally, team leaders’ influence regulation was positively correlated with individual-level job satisfaction and unrelated to two aspects of job performance. These results provided preliminary support for the hypothesized associations between the study variables.

**Table 1:** Descriptive statistics and intercorrelations among the study variables.

Variable	M	SD	1	2	3	4	5	6	7	8
*Level 1*										
1. Team member’s influence regulation	71.56	12.81	^-^							
2. Team member’s psychological capital	51.89	7.84	.08	-						
3. Team member’s job satisfaction	45.94	7.72	.23***	.37***	-					
4. Team member’s in-role performance	19.09	2.08	.07	.32***	.31***	-				
5. Team member’s creative performance	14.85	2.89	.17**	.59***	.35***	.25***	-			
*Level 2*										
6. Team leader’s influence regulation	78.68	5.87	.26***	-.01	.25***	.11	.11	-		
7. Team job satisfaction	14.00	2.87	.17**	.09	.26***	.18**	.19**	.21	-	
8. Team performance	13.00	1.60	.18**	.14*	.37***	.16**	.16**	.06	.30	-

*Note*. Spearman correlation coefficient is used. N = 309 individuals (level 1) in 34 groups (level 2), except for group-level correlations among team leader’s influence regulation, team job satisfaction and team performance (*N* = 34).

***p < .001;

**p < .01;

*p < .05.

To test Hypotheses 1–3 and 5, the hierarchical linear modeling (HLM) analyses were conducted in HLM 8.0. All variables were standardized before the analyses, and the restricted maximum likelihood method of estimation was applied. First, to confirm whether it was necessary to conduct the HLM procedure, we performed a series of three unconditional models with team members’ job satisfaction and two facets of job performance entered into these models as the outcome variables (level-1 model: *y*_*ij*_ = *β*_*0j*_ + *r*_*ij*_, level-2 model: *β*_*0j*_ = *γ*_*00*_ + *u*_*0j*_, mixed model: *y*_*ij*_ = *γ*_*00*_ + *u*_*0j*_ + *r*_*ij*_). The results of the one-way analyses of variance indicated statistically significant variability in team members’ job satisfaction (*χ*^*2*^(33) = 111.00, *p* < .001), in-role performance (*χ*^*2*^(33) = 99.72, *p* < .001), and creative performance (*χ*^*2*^(33) = 79.75, *p* < .001), thus supporting the use of the multilevel approach. Additionally, to assess the proportion of the total variance that can be explained by group membership, we calculated the interclass correlation coefficients (ICC) from the random-coefficient models [64]. The ICCs values were .20 for general job satisfaction, .14 for in-role performance, and .14 for creative performance.

In the next step, the multilevel relationships were investigated through three separate hierarchical linear models, with team members’ job satisfaction and job performance (i.e., in-role performance and creative performance) measured at the individual level as the outcome variables. In the constructed models, five predictors were taken into account. Team members’ influence regulation (DEI) and team members’ PsyCap (PSYCAP) were included as the individual level predictors (level-1 models: *y*_*ij*_ = *β*_*0j*_ + *β*_*1j*_(DEI) + *β*_*2j*_(PSYCAP) + *r*_*ij*_). Moreover, team leader’s influence regulation (DEILeader), teams’ job satisfaction (TeamJobSatisfaction) and team performance (TeamPerformance) were added as the group level predictors (level-2 models: *β*_*0j*_ = *γ*_*00*_ + *γ*_*01*_(DEILeader) *+ γ*_*02*_(TeamPerformance) *+ γ*_*03*_(TeamJobSatisfaction) *+ u*_*0j*_; *β*_*1j*_ = *γ*_*10*_; *β*_*2j*_ = *γ*_*20*_). The mixed models, incorporating both the level-1 and level-2 predictors, were: *y*_*ij*_ = *γ*_*00*_ + *γ*_*01*_(DEILeader) *+ γ*_*02*_(TeamPerformance) *+ γ*_*03*_(TeamJobSatisfaction) + *γ*_*10*_(DEI) + *γ*_*20*_(PSYCAP) + *u*_*0j*_+ *r*_*ij*_. In addition, to estimate the overall variance explanation, the pseudo R-squared was calculated separately for each model [65].

Table 2 presents the summarized results of the final multilevel analyses used to test the hypotheses. At the individual level, team members’ PsyCap appeared to positively predict individual-level job satisfaction and all forms of job performance, providing full support for Hypothesis 1. The strongest associations were observed between PsyCap and creative performance, followed by in-role performance and job satisfaction. Contrary to expectations, no significant associations were found between team members’ influence regulation and individual-level job satisfaction and the two facets of job performance (Hypothesis 2). In addition, support for Hypotheses 3 was not found because the leader’s influence regulation was unrelated to individual-level general job satisfaction and the two job performance variables analyzed in the present study. Finally, team job satisfaction was linked to team members’ job satisfaction, whereas team performance was positively associated with team members’ creative performance. These results partially support Hypothesis 5 referring to the cross-level relations between the outcome variables.

**Table 2:** Results of multilevel analyses predicting team member’s job satisfaction and job performance.

Variables	Team member’s job satisfaction		Team member’s in-role performance		Team member’s creative performance	
	*b*	SE	*b*	SE	*b*	SE
*Level 1*						
Intercept	-.02	.06	.07	.08	-.01	.06
Team member’s influence regulation	.10	.05	-.02	.04	.08	.05
Team member’s psychological capital	.28***	.05	.33***	.06	.55***	.05
*Level 2*						
Team leader’s influence regulation	.08	.08	.04	.11	.13	.07
Team job satisfaction	.25***	.06	-.01	.07	-.02	.04
Team performance	.10	.06	.11	.06	.11*	.05
*Number of estimated parameters*	2		2		2	
*Deviance*	801.88		804.52		739.86	
*Pseudo R* ^*2*^	.06		.12		.31	

*Note*. N (Level 2) = 34, N (Level 1) = 309. All variables were standardized.

Finally, to test Hypothesis 5, two simple linear regression models were developed with team leaders’ influence regulation as the predictor and team job satisfaction and team performance as the criterion variables. The model for team job satisfaction was insignificant (*F*(1,33) = 1.86; *p* > .05), and team leaders’ influence regulation was not a significant predictor of team job satisfaction (*β* = .03; *p* > .05). Similarly, the model for team performance was also insignificant (*F*(1.33) = .40; *p* > .05), with insignificant team leaders’ influence regulation as a predictor (*β* = -.02; *p* > .05).

## Discussion

The current study adopted a positive approach to job attitudes and organizational behavior in order to develop a more comprehensive research model involving individual and group factors that enhance job satisfaction and job performance among team members. More specifically, this study replicated and extended previous research on Fredrickson’s [11] broaden-and-build theory of positive emotions and their positive consequences and positive organizational behavior [9,52] by examining how the caravan of resources [54], namely, the bundle of PsyCap and influence regulation, is related to job satisfaction and two facets of job performance at the individual and group levels.

The findings provided mixed support for the proposed multilevel model. Consistent with the previous empirical evidence on the associations between PsyCap and employee job attitudes [9], team members’ PsyCap positively predicted job satisfaction at the individual level. Moreover, in line with prior research [e.g., 18,19], the multilevel analysis showed positive relationships between PsyCap and different facets of individual job performance, ranging from strong for creative performance to moderate for in-role performance. The strong links between PsyCap and performance-related outcomes are probably attributable to the proactive nature of creative performance. Simultaneously, in line with broaden-and-build theory, greater positivity expressed as PsyCap could increase the tendency to engage in this type of positive organizational behavior [66]. On a more general level, the results confirmed that PsyCap plays a crucial role in predicting job performance and behavioral and attitudinal outcomes in the workplace [9]. Although previous research suggested some cultural differences in manifestations of PsyCap [67–69], the present study revealed similarity in the importance of PsyCap in predicting job satisfaction and job performance in the Polish culture.

At the individual level, the correlation analysis confirmed that team members’ influence regulation was positively related to their job satisfaction and creative performance. However, the multilevel analysis showed no significant associations between team members’ influence regulation and employee job satisfaction and the two aspects of job performance. This result implies that the significant correlations were mainly based on aggregations. In addition, our findings also suggest that influence regulation might be seen as a relational construct. Consequently, it might better predict the quality and dynamics of interpersonal relationships between a team leader and team members than socially desirable job attitudes and different types of job performance. Therefore, future research should examine the possible linkages between influence regulation and relational factors, reflecting the process of shaping interpersonal relationships and the social influence exerted by an individual in team and organizational settings. Because influence regulation is relatively easy to observe and mainly expressed during the process of team communication [26], it would be worthwhile to investigate its associations with factors reflecting communication in face-to-face interactions in the workplace, such as interpersonal communication skills or communication patterns among team members. The weak or nonsignificant associations between influence regulation and job satisfaction and job performance might also result from treating influence regulation as a state-like construct, similar to PsyCap [20]. However, because the concept of influence regulation is derived from the situational theories of leadership and management [28], it might be even more changeable over time than PsyCap.

Unexpectedly, cross-level analyses demonstrated that the team leader’s influence regulation did not significantly predict individual-level job satisfaction and job performance. Similarly, at the organizational level, the team leader’s influence regulation was found to be unrelated to team job satisfaction and team performance. These results suggest that conscientious influence regulation undertaken by team leaders might not be the key factor determining positive outcomes in work teams. Given that the team leader’s influence regulation reflects positive aspects of organizational leadership, its relationships with team job satisfaction and team performance could be weaker than the influence of detrimental characteristics of the team leader, encompassed, for instance, by the constructs of abusive supervision [70] and destructive leadership in the workplace [71]. Our findings might also reflect the characteristics of the research sample, which consisted of formal leaders from large, well-established organizations who probably engage in influence regulation to a lesser extent than their informal counterparts. Most supervisors and managers might perceive sharing power through influence regulation as unimportant or unnecessary for enhancing team effectiveness. Moreover, team members could evaluate their formal leaders based on criteria other than an individual tendency to regulate their own influence.

Contrary to the expectations, we provided only partial support for the hypothesis concerning positive relationships between job satisfaction and job performance at both levels of analysis. We found that team job satisfaction was positively related to team member’s job satisfaction, whereas team performance was positively linked to team member’s creative performance. These findings might stem from the type of measurement used at the individual and group levels. Firstly, team job satisfaction and team performance were assessed by a team leader, who might evaluate them differently than team members. In addition, we applied the multidimensional conceptualization of job performance at the individual level and simultaneously the unidimensional one at the team level, which resulted in employing distinct measures of job performance at both levels of analysis.

## Strength, limitations, and future directions

The present study has several strengths. First, it makes an important contribution to studies on positive organizational behavior by referring to the construct of influence regulation. Using this concept extends the knowledge about positive psychological resources predicting job satisfaction and job performance, and it provides additional insight into how team leaders and team members might more effectively cooperate and share influence. With regard to positive organizational psychology, reconceptualizing the concept of positive psychological resources by deriving influence regulation from it might help to integrate different trends in research on social influence in organizations and its consequences. Considering the phenomenon of influence regulation with regard to positive psychology broadens our understanding of the associations between positive states and positive organizational outcomes in work teams. In particular, our findings shed new light on positive psychological resources that might help to identify not only flourishing employees, but also flourishing teams [72]. Additionally, because the present study used a multilevel approach, it offers the potential to better understand the cross-level associations among PsyCap, influence regulation, job satisfaction, and various facets of job performance in team leaders and team members. Another strength of this study is the diverse research sample, which includes working adults from different lines of business located in Poland. In line with the results of prior meta-analyses [9,73], this study suggested the similarity in manifestations of PsyCap in the form of positive work attitudes and organizational behavior in different cultures. However, further cross-cultural research on the behavioral and attitudinal consequences of PsyCap is needed.

However, the present study has some limitations. Firstly, although the concept of influence regulation offers the potential to integrate the organizational literature, it requires further empirical verification. In particular, future research should focus on a broader range of potential antecedents and consequences of influence regulation in the workplace. A second limitation is related to the research sample, which consists of team leaders and team members employed in restructured enterprises. Because organizations usually restructure in order to adapt to a crisis and more effectively respond to a dynamic situation [74], the uncertainty accompanying an organizational crisis might have affected the results of our study. Given that an organizational crisis influences the functioning of leaders and team members in the workplace [5], it would be worthwhile to test the associations between the study variables in different organizations operating in a more stable environment. Moreover, the sample size in this study was rather small; a total of 34 team leaders were included, and some work teams had only two members. An additional characteristic of the sample that might be seen as a limitation is related to the imbalanced gender ratio resulting from the predominance of women. Furthermore, we relied solely on self-report measures, which unables to conclude about the influence of positive psychological resources on work outcomes. Relying on self-reported data may also yield biased results due to the participants’ desire to manage their image or their fear of the research results being used to make organizational decisions that may have negative consequences. Also, the danger of a common method bias has to be considered when a set of questionnaires is used [75]. Hence, future research should include multi-wave and multi-source data collection to prevent the aforementioned problems.

Another limitation of this study might stem from the method of assessing influence regulation, job satisfaction, and job performance at the group level. Considering that team leaders’ influence regulation, team job satisfaction, and team performance were evaluated from the team leader’s perspective, they reflect only the team leader’s perceptions of his/her own influence regulation and team outcomes. Nevertheless, because influence regulation is treated as an individual ability to adequately exert influence on others in the workplace [26], team leaders and team members might understand it in different ways, depending on the perceived consequences for a leader, a particular employee, or a team as a whole. The present study focused on job satisfaction and performance among team members; therefore, the team member’s perspective would be more appropriate for testing the associations at the organizational level. In addition, because only the general level of team performance was measured at the organizational level, the differences in manifestations of team leaders’ influence regulation in the form of team outcomes were not captured. A multisource perspective in research on team performance that includes objective and subjective indices is highly recommended [76]. Therefore, further studies should include different facets of team performance at the individual and group levels of analysis, which would be measured both from the team member’s and team leader’s perspectives. In addition, to reduce same-source bias, we should also consider objective ratings of employee job performance and team performance included in company records and based on measurable and observable criteria [77].

Finally, the proposed research model might be viewed as rather noncomplex, as the number of independent and dependent variables on both the individual and team levels is limited. However, we think of this limitation as a simultaneous strength. To our knowledge, this is the first study to examine the relationships of PsyCap and influence regulation with job satisfaction and job performance from a multilevel perspective. Because it is a preliminary study, we intended to propose a simple model, but with salient variables, in order not to blur any hypothesized relationships and their explanations. Therefore, future research should broaden the model and include other variables, including moderators, mediators, and control variables [78] in order to build on the initial theoretical model.

## Conclusions and practical implications

In summary, using the broaden-and-build theory of positive emotions and positive organizational behavior framework, we investigated the relationships between influence regulation, PsyCap, job satisfaction, and job performance in work teams. Based on data collected from a sample of 34 team leaders and 304 team members, we conducted a multilevel hierarchical analysis. Our results confirmed that team members’ PsyCap is positively related to individual-level job satisfaction and two facets of job performance, including creative performance and in-role performance. The results of correlation analysis indicated that team members’ influence regulation was positively associated with employee job satisfaction and creative performance. However, the multilevel analysis demonstrated that team members’ influence regulation and team leader’s influence regulation were unrelated to both individual- and group-level job satisfaction and job performance. Our findings suggest the limited importance of influence regulation in predicting socially desirable work attitudes and positive outcomes in work teams, which might stem from its relational nature. This counterintuitive result may mean that in some circumstances, the leader’s influence is not important for team functioning, and it confirms some research results showing no relation between the leader and team performance. This may suggest that team members’ individual ability to interact, cooperate, exchange information, and adapt to work requirements are more important than the leader’s behavior. Therefore, our results highlight the fact that it is necessary for researchers to focus on both the individual and team levels of influence, due to the construct’s complex social nature.

Regarding the implications of the obtained findings, we feel it is worthwhile to consider applying them in business practice to form the subjective relationships inside the organization. Being a subject in a relationship implies the ability to make choices [79], which manifests as more control over action and decision-making. Individuals’ execution of professional obligations increases, and job performance improves as a consequence. At the same time, being a subject in a team corresponds to a feeling of community and a sense of partnership in action [80]. Therefore, team members bear responsibility for changes that occur as a result of joint actions. Actions of all organization members aimed at activating subjectivity will enhance individual activity, increase motivation to exert greater effort at work, and support creative activities undertaken by individuals, including the development of interpersonal relations. It means in the practice that managers should be aware that it is still not clear whether there is any link between managers’ behavior and employees’ productivity and satisfaction [81], and therefore more attention in the process of preparing managers for work through training and self-development should be paid to building partnerships in the team, the ability to provide space for influence and decision-making by empowered employees.

## Supporting information

S1 File The decision of the Ethics Committee of the University of Valencia. (PDF)

S2 File Methods used to collect data from team leaders. (PDF)

S3 File Methods used to collect data from team members. (PDF)
