# Supplementary material for: The relationships of psychological capital and influence regulation with job satisfaction and job performance
Source: PLoS One. 2022 Aug 9;17(8):e0272412. doi: 10.1371/journal.pone.0272412 (PMC9362931; doi:10.1371/journal.pone.0272412)
Supplement: S1 File — (PDF) [file pone.0272412.s001.pdf]

VNIVERSITAT
DE VALÈNCIA
Vicerectorat d'Investigació i Política Científica

D. Màrius V. Fuentes Ferrer, Profesor Titular del departamento de Biología Celular y Parasitología, y Presidente del Comité Ético de Investigación en Humanos de la Comisión de Ética en Investigación Experimental de la Universitat de València,

CERTIFICA:

Que el Comité Ético de Investigación en Humanos, en la reunión celebrada el día 10 de diciembre de 2012, una vez estudiado el proyecto de investigación titulado:
"Las características del puesto de trabajo y las prácticas de recursos humanos como antecedentes de un bienestar laboral sostenible en las distintas etapas de la carrera", número de procedimiento H1354632059685,
cuyo investigador responsable es D. José Mª Peiró Silla,
ha acordado informar favorablemente el mismo dado que se respetan los principios fundamentales establecidos en la Declaración de Helsinki, en el Convenio del Consejo de Europa relativo a los derechos humanos y cumple los requisitos establecidos en la legislación española en el ámbito de la investigación biomédica, la protección de datos de carácter personal y la bioética.

Y para que conste, se firma el presente certificado en Valencia, a once de diciembre de dos mil doce.

UNIVERSITAT DE VALÈNCIA

Carrer: Blasco Ibáñez, 13
VALENCIA 46071

Telèfon: (96) 386 41 09
Fax: (96) 398 32 21
e-mail: vicerec.investigacio@uv.es
