# Supplementary material for: The relationships of psychological capital and influence regulation with job satisfaction and job performance
Source: PLoS One. 2022 Aug 9;17(8):e0272412. doi: 10.1371/journal.pone.0272412 (PMC9362931; doi:10.1371/journal.pone.0272412)
Supplement: S2 File — (PDF) [file pone.0272412.s002.pdf]

Influence Regulation and Deinfluentization Scale (*DEI-beh*)

Source: Kożusznik B, Pollak A, Adamek D, Grabowski D. Development and Validation of the Influence Regulation and Deinfluentization Scale (DEI-beh). Polish Journal of Applied Psychology [Internet]. Walter de Gruyter GmbH; 2015 Dec 1;13(4):91-108. Available from: http://dx.doi.org/10.1515/pjap-2015-0044

Indicate to what extent you agree with the following statements about yourself. The five response choices are: 1 = never, 2 = rarely, 3 = sometimes, 4 = often, 5 = always.

1.	I am able to remain silent although I could take part in a conversation.	1 2 3 4 5
2.	I am able to wait through a break in a conversation.	1 2 3 4 5
3.	I am able to encourage others to express their opinions.	1 2 3 4 5
4.	I am able to abstain from commenting.	1 2 3 4 5
5.	I care about maintaining proper space in contact with others.	1 2 3 4 5
6.	I can diminish my own importance in a conversation.	1 2 3 4 5
7.	I can lower my gaze not to cause embarrassment to an interlocutor.	1 2 3 4 5
8.	I am able to change a conversation topic without causing embarrassment to an interlocutor.	1 2 3 4 5
9.	I keep calm when my talk is interrupted.	1 2 3 4 5
10.	I am able to acknowledge my mistakes.	1 2 3 4 5
11.	I try to respect private, intimate space between persons (e.g. I restrain from involuntary touching others during a conversation)	1 2 3 4 5
12.	I can move over to make room for someone.	1 2 3 4 5
13.	I keep eye contact with an interlocutor.	1 2 3 4 5
14.	I arrange space to make it comfortable for participants in a conversation.	1 2 3 4 5
15.	I approve of other people’s ideas.	1 2 3 4 5
16.	I can backtrack.	1 2 3 4 5
17.	I calm down noise and talking that make it impossible for others to express their opinions.	1 2 3 4 5
18.	I wait until others finish their utterance.	1 2 3 4 5
19.	I care about appropriate and convenient distance between people.	1 2 3 4 5
20.	I keep a serene facial expression despite a difficult situation.	1 2 3 4 5

Perceived group performance scale (Team performance)

Source: Jehn KA, Northcraft GB, Neale MA. (1999). Why Differences Make a Difference: A Field Study of Diversity, Conflict, and Performance in Workgroups. Adm Sci Q 1999;44: 741-63.

Please answer the following questions about **YOUR UNIT WORKERS IN GENERAL**, using the response scale offered below:

Definitely bad	Bad	Average	Good	Definitely good
1	2	3	4	5

1.	How well do you think your work team performs?	1 2 3 4 5
2.	What is the quality of the work done by your work unit?	1 2 3 4 5
3.	What was the level of goal achievement of your work unit last year?	1 2 3 4 5

General Job Satisfaction

Source: European research project on sustainable wellbeing at work (BELASOS: PSI 2015-64862-R (MINECO FEDER) *Job characteristics and human resource practices as antecedents of sustainable wellbeing at work at different career stages.* Coordinating unit - University of Valencia).

Please answer the following questions about **YOUR UNIT WORKERS IN GENERAL**, using the response scale offered below:

Very dissatisfied	Dissatisfied	Moderately dissatisfied	Neither satisfied nor dissatisfied	Moderately satisfied	Satisfied	Very satisfied
1	2	3	4	5	6	7

In general, how satisfied are the workers in your unit ...

1.	with the job they do?	1 2 3 4 5 6 7
2.	with the social climate and personal relationships in the unit?	1 2 3 4 5 6 7
3.	with the pay and compensation they receive for their work?	1 2 3 4 5 6 7

Demographic data

Age	☐ under 35 years old ☐ 35-50 years old ☐ over 50 years old
Sex	☐ Male ☐ Female
Educational level:	☐ Compulsory education (primary school)<br>☐ vocational school<br>☐ Secondary education (high school)<br>☐ University degree (master’s degree)
