# Supplementary material for: The relationships of psychological capital and influence regulation with job satisfaction and job performance
Source: PLoS One. 2022 Aug 9;17(8):e0272412. doi: 10.1371/journal.pone.0272412 (PMC9362931; doi:10.1371/journal.pone.0272412)
Supplement: S3 File — (PDF) [file pone.0272412.s003.pdf]

Psychological Capital Questionnaire Research Permission

Fred Luthans, Bruce J. Avolio, and James B. Avey

Prepared on October 30, 2019 for:

Mateusz Paliga

You completed your evaluation at 1:30 am EDT on October 30, 2019.

mind garden

Copyright © 2007 Fred Luthans, Bruce J. Avolio, and James B. Avey. All rights reserved in all medium. Published by Mind Garden, Inc. www.mindgarden.com

To whom it may concern,

This letter is to grant permission for Mateusz Paliga to use the following copyright material:

Instrument: ***Psychological Capital (PsyCap) Questionnaire (PCQ)***

Authors: ***Fred Luthans, Bruce J. Avolio & James B. Avey.***

Copyright: ***"Copyright © 2007 Psychological Capital (PsyCap) Questionnaire (PCQ) Fred L. Luthans, Bruce J. Avolio & James B. Avey. All rights reserved in all medium."***

for his/her thesis/dissertation research.

Three sample items from this instrument may be reproduced for inclusion in a proposal, thesis, or dissertation.

The entire instrument may not be included or reproduced at any time in any other published material.

Sincerely,

Mind Garden, Inc.
www.mindgarden.com

Copyright © 2007 Fred Luthans, Bruce J. Avolio, and James B. Avey. All rights reserved in all medium. Published by Mind Garden, Inc. www.mindgarden.com

Psychological Capital Questionnaire (PCQ-12, sample questions)

Copyright © 2007 Psychological Capital (PsyCap) Questionnaire (PCQ) Fred L. Luthans, Bruce J. Avolio & James B. Avey. All right reserved in all medium.

Research permission was granted to Mateusz Paliga on October 30, 2019, by Mind Garden, Inc.

Below are statements that describe how you may think about yourself right now. Use the following scale to indicate your level of agreement or disagreement with each statement:

**Strongly disagree**	**Disagree**	**Somewhat disagree**	**Somewhat agree**	**Agree**	**Strongly agree**
1	2	3	4	5	6

1.	I feel confident analyzing a long-term problem to find a solution.	1 2 3 4 5 6
2.	If I should find myself in a jam at work, I could think of many ways to get out of it.	1 2 3 4 5 6
3.	When I have a setback at work, I have trouble recovering from it, moving on.	1 2 3 4 5 6

Influence Regulation and Deinfluentization Scale (*DEI-beh*)

Source: Kożusznik B, Pollak A, Adamek D, Grabowski D. Development and Validation of the Influence Regulation and Deinfluentization Scale (DEI-beh). Polish Journal of Applied Psychology [Internet]. Walter de Gruyter GmbH; 2015 Dec 1;13(4):91-108. Available from: http://dx.doi.org/10.1515/pjap-2015-0044

Indicate to what extent you agree with the following statements about yourself. The five response choices are: 1 = never, 2 = rarely, 3 = sometimes, 4 = often, 5 = always.

1.	I am able to remain silent although I could take part in a conversation.	1 2 3 4 5
2.	I am able to wait through a break in a conversation.	1 2 3 4 5
3.	I am able to encourage others to express their opinions.	1 2 3 4 5
4.	I am able to abstain from commenting.	1 2 3 4 5
5.	I care about maintaining proper space in contact with others.	1 2 3 4 5
6.	I can diminish my own importance in a conversation.	1 2 3 4 5
7.	I can lower my gaze not to cause embarrassment to an interlocutor.	1 2 3 4 5
8.	I am able to change a conversation topic without causing embarrassment to an interlocutor.	1 2 3 4 5
9.	I keep calm when my talk is interrupted.	1 2 3 4 5
10.	I am able to acknowledge my mistakes.	1 2 3 4 5
11.	I try to respect private, intimate space between persons (e.g. I restrain from involuntary touching others during a conversation)	1 2 3 4 5
12.	I can move over to make room for someone.	1 2 3 4 5
13.	I keep eye contact with an interlocutor.	1 2 3 4 5
14.	I arrange space to make it comfortable for participants in a conversation.	1 2 3 4 5
15.	I approve of other people's ideas.	1 2 3 4 5
16.	I can backtrack.	1 2 3 4 5
17.	I calm down noise and talking that make it impossible for others to express their opinions.	1 2 3 4 5
18.	I wait until others finish their utterance.	1 2 3 4 5
19.	I care about appropriate and convenient distance between people.	1 2 3 4 5
20.	I keep a serene facial expression despite a difficult situation.	1 2 3 4 5

In-role Performance Scale

Source: Williams LJ, Anderson SE. Job Satisfaction and Organizational Commitment as Predictors of Organizational Citizenship and In-Role Behaviors. Journal of Management [Internet]. SAGE Publications; 1991 Sep;17(3):601-17. Available from: http://dx.doi.org/10.1177/014920639101700305

Please, indicate your degree of agreement or disagreement with each statement, using the following response scale:

Strongly disagree	Moderately disagree	Somewhat disagree	Neither agree nor disagree	Somewhat agree	Moderately agree	Strongly agree
1	2	3	4	5	6	7

1.	I adequately complete assigned duties	1 2 3 4 5 6 7
2.	I fulfill responsibilities specified in job description	1 2 3 4 5 6 7
3.	I perform tasks that are expected of mine	1 2 3 4 5 6 7

Creative Performance Scale

Source: Oldham GR, Cummings A. Employee Creativity: Personal And Contextual Factors At Work. Acad Manage J [Internet]. Academy of Management; 1996 Jun 1;39(3):607-34. Available from: http://dx.doi.org/10.2307/256657

Please, indicate your degree of agreement or disagreement with each statement, using the following response scale:

Strongly disagree	Moderately disagree	Somewhat disagree	Neither agree nor disagree	Somewhat agree	Moderately agree	Strongly agree
1	2	3	4	5	6	7

1.	I am practical at work. I develop ideas that are useful to the organization	1 2 3 4 5 6 7
2.	I am flexible at my job. I adapt in a creative way the resources available for me	1 2 3 4 5 6 7
3.	I am creative at my job. I develop original ideas for my organization	1 2 3 4 5 6 7

Job Satisfaction Scale

Source: Cooper CL, Rout U, Faragher B. Mental health, job satisfaction, and job stress among general practitioners. BMJ [Internet]. BMJ; 1989 Feb 11;298(6670):366-70. Available from: http://dx.doi.org/10.1136/bmj.298.6670.366

Below, indicate to what extent you feel satisfied with each of the aspects presented in the following sentences, using the following response scale:

**Very dissatisfied**	**Dissatisfied**	**Moderately dissatisfied**	**Neither satisfied nor dissatisfied**	**Moderately satisfied**	**Satisfied**	**Very satisfied**
1	2	3	4	5	6	7

1.	Freedom to choose your own method of working	1 2 3 4 5 6 7
2.	Recognition you get for your good work	1 2 3 4 5 6 7
3.	Amount of responsibility you are given	1 2 3 4 5 6 7
4.	Opportunity to use your ability	1 2 3 4 5 6 7
5.	Amount of variety in your job	1 2 3 4 5 6 7
6.	Physical working conditions	1 2 3 4 5 6 7
7.	Your fellow workers	1 2 3 4 5 6 7
8.	Your rate of pay	1 2 3 4 5 6 7
9.	Your hours of work	1 2 3 4 5 6 7

Demographic data

Age	☐ under 35 years old ☐ 35-50 years old ☐ over 50 years old
Sex	☐ Male ☐ Female
Educational level:	☐ Compulsory education (primary school)<br>☐ vocational school<br>☐ Secondary education (high school)<br>☐ University degree (master’s degree)
